# Case Report: NGS-guided rapid diagnosis of tuberculous otitis media—a rare case of dual-site *Mycobacterium tuberculosis* infection

**DOI:** 10.3389/fmed.2025.1734666

**Published:** 2026-01-08

**Authors:** Yang Gao, Xinlei Wang, Yi Cheng, Shengxin Ye, Xin Dong, Chi Zhu

**Affiliations:** 1 Department of Tuberculosis, Heze Infectious Disease Hospital, Heze, China; 2 Department of Clinical Laboratory, Hangzhou Adicon Medical Laboratory Co., Ltd., Hangzhou, China; 3 Department of Respiratory and Critical Care Medicine, Affiliated Changshu Hospital of Nantong University, Changshu, China

**Keywords:** disseminated tuberculosis, extrapulmonary tuberculosis, infectious diseases, next-generation sequencing, tuberculous otitis media

## Abstract

**Background:**

Tuberculous otitis media (TOM) is an exceptionally rare form of extrapulmonary tuberculosis that was usually diagnosed only after long-standing ear discharge or profound hearing loss. This case reported a young man in whom deafness was the sentinel event leading to the discovery of pulmonary tuberculosis and molecular confirmation of concurrent TOM.

**Case presentation:**

A 23-year-old male presented with bilateral, progressive hearing loss that had been labeled “chronic suppurative otitis media” by local clinics. Persistent constitutional symptoms prompted chest imaging that revealed bilateral cavitary infiltrates. Broncho-alveolar lavage metagenomic next-generation sequencing identified *Mycobacterium tuberculosis* complex (MTBC). After transfer to our tuberculosis center, targeted NGS of serous middle-ear fluid detected MTBC; the isolate carried an rpsL K43R mutation conferring streptomycin resistance, identical to the pulmonary strain. Standard four-drug anti-tuberculosis therapy was initiated; within 4 weeks, cough and fever resolved, inflammatory markers normalized, and the pulmonary cavity showed reduction in size compared to baseline.

**Conclusion:**

This case highlights that unexplained hearing loss may serve as an early indicator of disseminated tuberculosis. High-throughput sequencing of aural discharge enables rapid diagnosis of TOM, facilitates resistance-guided treatment, and helps trace the pathways of pathogen transmission.

## 1 Introduction

Tuberculosis (TB) remains the leading cause of infectious mortality among adults worldwide, yet its extrapulmonary manifestations are the most challenging to diagnose and manage (1, 2). In China, extrapulmonary TB accounted for 24.6% of all diseases during 2020–2021 (3). The most common anatomical sites affected by extrapulmonary TB were the lymph nodes, pleura, bones and joints, urogenital tract, and meninges (4, 5). Tuberculous otitis media (TOM) was an uncommon but increasingly recognized extrapulmonary manifestation of MTB, accounting for 0.1% of all TB cases and otitis media (3, 6). Due to its clinical and otoscopic features closely resembling those of chronic suppurative otitis media (CSOM), the TOM was usually mistaken for CSOM or noise-induced hearing loss, resulting in inappropriate antibiotics and irreversible sensorineural hearing loss or facial nerve paralysis (7). Bilateral TOM was even rarer, and its simultaneous occurrence with active pulmonary TB has been reported only sporadically (8).

The sluggish growth of MTB, intricate operational processes, and stringent biosafety requirements associated with MTB render culture-based detection methods inadequate for rapid clinical diagnosis (9). The availability of metagenomic and targeted next-generation sequencing (NGS) enables the direct detection of pathogen nucleic acids from body fluids within 24–48 h (10). These platforms circumvent the prolonged culture period required for *Mycobacterium tuberculosis* complex (MTBC) and concurrently deliver drug-resistance information, making them particularly suitable for paucibacillary specimens such as middle-ear effusions (11, 12). To date, few reports have employed NGS to identify MTBC in aural discharge, and presented deafness as the sentinel symptom leading to the discovery of pulmonary TB.

This case report describes a patient who presented with bilateral hearing loss that was refractory to standard antimicrobial therapy for presumed CSOM. Systemic review ultimately revealed concurrent pulmonary TB, and NGS of both BALF and ear discharge confirmed the same MTB resistance genotype. This case illustrates the diagnostic pitfalls of TOM and highlights the value of integrating chest imaging and molecular diagnostics in the evaluation of persistent otitis media.

## 2 Case presentation

A 23-year-old male farmer with a four-year history of smoking, averaging approximately 10 cigarettes per day was transferred to our hospital (Tuberculosis Department, Heze Infectious Disease Hospital, China) on 4 September 2025, following a 3-month history of productive cough, low-grade fever, night sweats, weight loss (10 kg), and progressive bilateral hearing loss over the past 2 months. Empirical anti-infective treatment with Cephalosporin drugs for 7 days for pneumonia and otitis media did not result in clinical improvement. The patient had been admitted to a local hospital’s otolaryngology department from 1 to 3 September 2025 with a presumptive diagnosis of severe community-acquired pneumonia and CSOM. At the local hospital, empirical moxifloxacin was initiated for presumed pneumonia, but symptoms persisted. Chest computed tomography (CT) revealed patchy and focal areas of increased attenuation with scattered cavities in both lungs. Bronchoalveolar lavage fluid (BALF) analyzed by metagenomic NGS revealed MTBC (3,474 reads) and *Candida albicans* (5,533 reads), prompting referral to our facility for specialized TB management.

**Figure 1:**
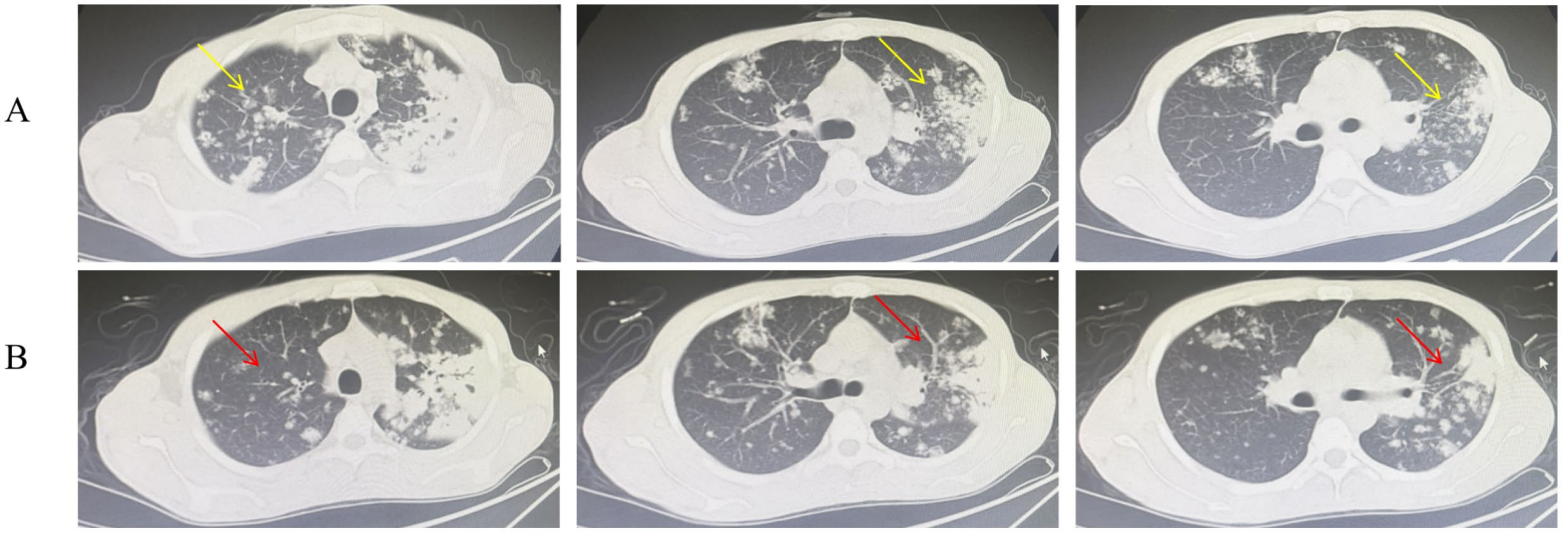
Chest CT scans before **(A)** and after **(B)** 4 weeks of anti-tuberculosis therapy. **(A)** Pre-treatment image shows apical-predominant infiltrates with a cavitary lesion (yellow arrow). **(B)** Post-treatment image shows resolution of infiltrates and reduction in cavity size (red arrow).

Upon admission, the patient presented with a cachectic appearance but was hemodynamically stable (temperature: 37.0 °C, blood pressure: 95/64 mmHg, heart rate: 68 beats per minute). The patient reported intermittent cough and sputum production, accompanied by occasional chest pain. Appetite remained intact, and gastrointestinal and urinary functions were within normal limits. On physical examination, the patient was a young male who was alert, with a height of 172 cm and a weight of 49 kg. Audiometric testing indicated bilateral mixed-type hearing loss. No superficial lymphadenopathy was detected on clinical examination. Bilateral lung auscultation revealed clear breath sounds with areas of decreased intensity; coarse breath sounds are present bilaterally, and moist rales are audible. A non-contrast chest CT scan revealed multiple patchy, flame-like, and nodular areas of high density distributed throughout both lung fields, predominantly in the upper lobes. Localized consolidation was observed in the left upper lobe, accompanied by an air bronchogram and a well-defined cavity measuring approximately 0.9 × 0.7 cm with smooth internal margins. The left hilar region appears prominent, while the trachea and bronchi remain patent, showing no evidence of stenosis or obstruction. The mediastinal structures are clearly delineated, with no space-occupying lesions or significantly enlarged lymph nodes identified. There is no bilateral pleural thickening, and no pleural effusion is present within the thoracic cavity (Figure 1A). Laboratory findings included Platelet count (PLT, 686 × 10^9^/L), anemia (hemoglobin [Hb]: 75 g/L), elevated inflammatory markers (leukocytosis: 15.65 × 10^9^/L, Neutrophil count: 12.17 × 10^9^/L, Erythrocyte sedimentation rate (ESR): 110 mm/h, Interleukin-6 [IL-6]: 51 pg./mL, fibrinogen [Fbg]: 5.39 g/L), and normal liver function (Alanine aminotransferase [ALT]: 28.3 U/L) (Table 1). Human Immunodeficiency Virus, Hepatitis B Virus, and Hepatitis C Virus serologies were negative. A positive result in the purified protein derivative (PPD) test suggests that the patient may have been infected with MTB. The patient’s sputum smear acid-fast staining yielded a 1 + result, and the Xpert MTB/rifampin (MTB/RIF) assay detected a low concentration of MTB nucleic acid susceptible to rifampicin. The BALF and ear discharge culture, which were sent to the laboratory on September 10, were reported as positive on October 16 and November 19, respectively. The drug susceptibility testing results confirmed that the isolate was resistant to streptomycin at both low (10 μg/mL) and high (100 μg/mL) concentrations. Moxifloxacin resistance was detected at both 1 μg/mL and 4 μg/mL concentrations. The isolate was phenotypically sensitive to all other first-line drugs tested, including isoniazid, rifampicin, and ethambutol. The mixed lymphocyte culture interferon-gamma release assay yielded a strongly positive result of 0.512 (normal range: 0–0.16), together with clinical symptoms, radiological findings, and positive microbiology, supported the diagnosis of active TB, prompting immediate initiation of anti-tuberculosis therapy.

**Table 1:** Laboratory test results for the patient upon admission and discharge.

Index	Admission	Discharge
WBC (×10^9^/L)	15.65↑	13.04
Neutrophil count (×10^9^/L)	12.17↑	9.81
Lymphocyte count (×10^9^/L)	1.77	1.93
Monocyte count (×10^9^/L)	1.46↑	1.06
RBC (×10^12^/L)	2.47	4.17
HGB (g/L)	75	127
PLT (×10^9^/L)	686	516
ESR (mm/h)	110↑	84↑
LDH (U/L)	208.2	230.4
PA (mg/dl)	95.1↓	126.90↓
ADA (U/L)	20.3	22.70
Hs-CRP (mg/L)	2.97	4.01
ALT (U/L)	28.3	13.4
GGT (U/L)	115.8↑	88.9↑
ALB (g/L)	32.5	39.6
CREA (umol/L)	54.4	53.1

WBC, white blood cell count; neutrophil count; lymphocyte count; monocyte count; RBC, red blood cell count; HGB, hemoglobin; PLT, platelet count; ESR, erythrocyte sedimentation rate; LDH, lactate dehydrogenase; PA, prealbumin; ADA, adenosine deaminase; Hs-CRP, high-sensitivity C-reactive protein; ALT, alanine aminotransferase; GGT, gamma-glutamyl transferase; ALB, albumin; CREA, creatinine.

**Figure 2:**
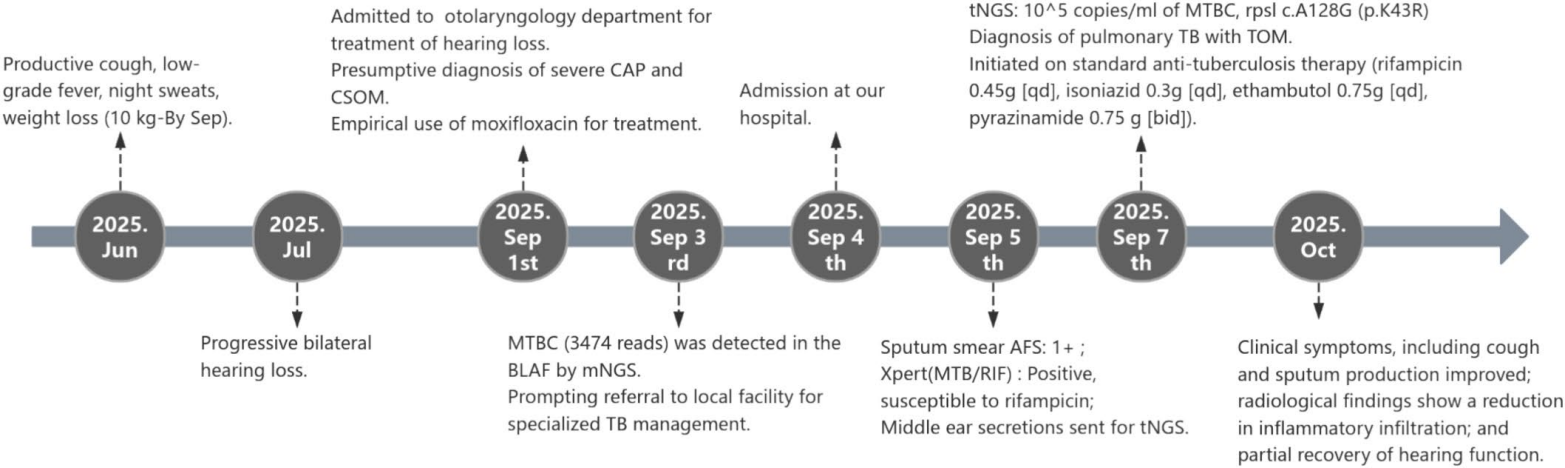
The diagnostic flowchart of the patient.

To investigate the possibility of dual-site tuberculosis involvement, we collected about 0.5 mL middle ear secretions on 5 September. tNGS (Salus Pro platform) detected 1*10^5 copies/ml of MTBC, indicating a high level of MTBC nucleic acid signals. Resistance analysis revealed a single, high-confidence resistance-conferring mutation: rpsl c.A128G (p.K43R) at 100% mutant frequency—strongly associated with streptomycin (S) resistance. All other screened loci (rpoB, katG, inhA, embB, pncA, gyrA/B, rrs) showed wild-type sequences, indicating full susceptibility to rifampicin, isoniazid, ethambutol, pyrazinamide and fluoroquinolones. Based on radiologic, microbiologic, and molecular evidence, a diagnosis of pulmonary tuberculosis with tuberculous otitis media was established. The diagnostic flowchart of the patient is presented in Figure 2.

To further evaluate whether the otic tuberculosis infection was attributable to disseminated spread from pulmonary foci, we performed an additional tNGS analysis on BALF. High concentrations (6 × 10^4^ copies/ml) of MTBC were detected in the BALF sample. The drug resistance gene profiles derived from BALF and ear secretions were completely concordant; integrating this genetic evidence with the patient’s respiratory symptoms 3 months ago and the onset of hearing loss a month later provides initial confirmation that the otic tuberculosis originated from pulmonary tuberculosis.

The patient was initiated on standard weight-based anti-tuberculosis therapy (rifampicin 0.45 g [qd], isoniazid 0.3 g [qd], ethambutol 0.75 g [qd], pyrazinamide 0.75 g [bid]) under directly observed treatment, with close monitoring of liver function and audiologic follow-up. After 4 weeks, inflammatory markers normalized (Table 1) and chest imaging (Figure 1B) showed early cavitation regression. The patient reported a noticeable reduction in cough and sputum production, along with improved hearing, compared to the pre-treatment state. A small amount of white, viscous sputum remains, with no visible hemoptysis, fever, or night sweats noted. No significant adverse reactions or toxic effects were observed during anti-tuberculosis therapy. The patient has been discharged and referred to a specialized otolaryngology hospital for further management of TOM.

## 3 Discussion

The clinical trajectory of this 23-year-old farmer encapsulates the diagnostic challenges posed by disseminated TB. What began as apparently straightforward progressive deafness, empirically ascribed to CSOM, ultimately unveiled bilateral cavitary pulmonary TB and concomitant TOM. The narrative highlights that unexplained, refractory hearing loss may herald systemic TB infection and demonstrates high-throughput sequencing of minute-volume ear fluid can simultaneously confirm TOM, delineate resistance, and illuminate the route of dissemination.

TOM is a rare but clinically significant extrapulmonary manifestation of MTB, accounting for approximately 0.04–0.9% of all CSOM cases (13). Despite its rarity, TOM carries substantial morbidity, particularly due to its non-specific presentation and propensity for diagnostic delay (14). The disease often caused painless otorrhea, early hearing loss, and multiple tympanic membrane perforations, though classic signs are now less evident due to prior antibiotic use (15). In a recent Chinese case series involving 23 patients, all presented with ear discharge and had conductive or mixed hearing loss, and 23.1% developed facial nerve palsy, underscoring the neuro-otologic severity of the disease (16). In the present case, bilateral progressive hearing loss was the sole presenting complaint for nearly 2 months; classic otorrhoea, otalgia, or tympanic perforation were absent. Such “paucisymptomatic” TOM was easily mislabelled as CSOM or noise-induced hearing loss. The prognosis was heavily dependent on early recognition; untreated cases may progress to labyrinthitis, facial paralysis, or intracranial complications such as tuberculous meningitis (17).

Diagnostic delay was the rule rather than the exception in TOM. In previous reports, the interval between initial examination and final diagnosis of TOM has been documented to extend up to 6 months or longer (6, 18). The proportion of cases diagnosed beyond 12 months was as high as 26.5% (19). The patient’s young age and rural occupation initially suggested a plausible but incorrect cause for bilateral hearing loss, highlighting the importance of considering TOM in refractory or progressive cases, especially with constitutional symptoms. Conventional work-ups rely on acid-fast bacilli staining, mycobacterial culture or temporal-bone biopsy, yet the middle-ear effusion is frequently paucibacillary and biopsy is invasive, yielding low sensitivity rates (20). By contrast, metagenomic or targeted NGS circumvent these bottlenecks: in the present case, only 0.5 mL of ear discharge obtained through atraumatic suction generated 1 × 10^5^ MTBC copies mL^−1^, and the entire laboratory turnaround time was 24 h. Importantly, the same run simultaneously delivered a high-confidence rpsL K43R streptomycin-resistance call at 100% allele frequency, information that culture would have required an additional 4–6 weeks to provide. These data corroborate recent study in which targeted NGS detected MTB with a sensitivity of 91.5% and specificity of 97.6% (12).

Tuberculosis bacteria were thought to reach the middle ear via the eustachian tube, disseminate hematogenously from other tuberculous foci, or be introduced directly through the external auditory canal following tympanic membrane perforation (21, 22). Radiologically, the bilateral, apical-predominant consolidations and cavities in this patient conform to the classic distribution of airborne tuberculosis. In post-primary tuberculosis, bacilli usually spread bronchogenically to the upper lobes, which offer a well-ventilated, high-oxygen environment, explaining the relative sparing of the lower lung zones. In contrast, if the pulmonary infection had originated from retrograde hematogenous dissemination of otic bacilli, a gravity-dependent, lower-lung predominance would be expected, as the lower lobes receive a greater proportion of cardiac output and are preferential sites for blood-borne pathogens. The minimal involvement of the lower lung fields therefore argues against the ear serving as the primary source. When imaging findings, the four-week interval between respiratory symptoms and hearing loss, and the identical rpsL K43R resistance genotype in both BLAF and middle-ear effusion were considered together, the most straightforward explanation was clonal hematogenous spread from pulmonary foci to the middle ea., not the reverse. We postulate that brief sub-clinical bacteraemia during cavity formation allowed bacilli to reach the labyrinthine artery and subsequently the promontorial mucosa, a pathway previously hypothesized but rarely substantiated by molecular data (21).

The report was subject to several constraints. Firstly, the absence of baseline audiometry prior to the onset of ear symptoms precludes a precise dissection of noise-induced versus tuberculosis-related hearing loss, rendering the quantification of reversible auditory improvement tentative. Secondly, although the identical resistance profiles strongly suggested clonal dissemination, we did not perform whole-genome sequencing of paired pulmonary and ear isolates; consequently, subtle single-nucleotide polymorphisms that might refine the transmission chain could have been missed. Thirdly, middle-ear fluid was obtained only once; serial NGS quantification of MTBC load over time would have strengthened the argument for molecular cure and provided pharmacodynamic insight. Finally, the patient was lost to otologic follow-up after week 4 so long-term audiometric outcomes, risk of relapse, or late labyrinthine complications remain unknown.

## 4 Conclusion

In conclusion, this case illustrates that TOM should enter the differential diagnosis of any patient with unexplained progressive deafness. Metagenomic or targeted NGS of minute volumes of aural discharge can rapidly establish the etiology, detect drug resistance and prevent irreversible neurological sequelae. We propose that ear, nose and throat clinics incorporate molecular TB testing into routine algorithm for refractory otitis, thereby shortening diagnostic delay and preserving both hearing and life.

Patient Perspective: The patient expressed profound relief that a definitive diagnosis was reached after months of uncertainty. He reported significant improvement in hearing and overall well-being after 4 weeks of therapy and remains committed to completing the full treatment course. He hopes this case will raise awareness among clinicians to consider TB in unusual presentations.

## Data Availability

The original contributions presented in the study are included in the article/supplementary material, further inquiries can be directed to the corresponding author.
